# Transcatheter Mitral Repair for Functional Mitral Regurgitation According to Left Ventricular Function: A Real-Life Propensity-Score Matched Study

**DOI:** 10.3390/jcm9061792

**Published:** 2020-06-09

**Authors:** Isaac Pascual, Fernando Carrasco-Chinchilla, Tomas Benito-Gonzalez, Chi Hion Li, Pablo Avanzas, Luis Nombela-Franco, Manuel Pan, Ana Serrador Frutos, Xavier Freixa, Ramiro Trillo-Nouche, Rosa A. Hernández-Antolín, Leire Andraka Ikazuriaga, Ignacio Cruz-Gonzalez, Jose R. López-Mínguez, Jose L. Diez, Alberto Berenguer-Jofresa, Juan Sanchis, Valeriano Ruiz-Quevedo, Cristobal Urbano-Carrillo, Juan F. Oteo Dominguez, Maria R. Ortas-Nadal, Eduardo Molina Navarro, Xavier Carrillo, Juan H. Alonso-Briales, Felipe Fernández-Vázquez, Luis Asmarats Serra, Daniel Hernandez-Vaquero, Pilar Jimenez-Quevedo, Dolores Mesa, Tania Rodríguez-Gabella, Ander Regueiro, Amparo Martinez Monzonís, Luisa Salido Tahoces, Lara Ruiz Gomez, Blanca Trejo-Velasco, Victor M. Becerra-Muñoz, Carmen Garrote-Coloma, Estafanía Fernández Peregrina, Rebeca Lorca, Jose A. De Agustín, Miguel Romero, Ignacio J. Amat-Santos, Manel Sabaté, Ana B. Cid Alvarez, Jose M. Hernandez-Garcia, Javier Gualis, Dabit Arzamendi, Cesar Moris, Gabriela Tirado-Conte, Angel Sánchez-Recalde, Rodrigo Estevez-Loureiro

**Affiliations:** 1 Heart Area, Asturias Central University Hospital, University of Oviedo, Instituto Investigación Sanitaria Principado de Asturias (ISPA), 33011 Oviedo, Spain; ipascua@live.com (I.P.); avanzas@gmail.com (P.A.); lorcarebeca@gmail.com (R.L.); cesarmoris@gmail.com (C.M.); 2 Cardiology Department, Virgen de la Victoria University Hospital, Instituto de Investigación Biomédica de Málaga (IBIMA), University of Málaga, CIBERCV, 29010 Málaga, Spain; fernandocarrascochinchilla@gmail.com (F.C.-C.); juanhalonso62@gmail.com (J.H.A.-B.); vmbecerram@gmail.com (V.M.B.-M.); josemaria2509@gmail.com (J.M.H.-G.); 3 Cardiology Department. University Hospital of León, 24008 León, Spain; tomasbenito@outlook.com (T.B.-G.); ffernandez@secardiologia.es (F.F.-V.); cgarrote@saludcastillayleon.es (C.G.-C.); javgua@hotmail.com (J.G.); 4 Cardiology Department, Santa Creu i Sant Pau Hospital, 08041 Barcelona, Spain; ch.pedroli@gmail.com (C.H.L.); LAsmarats@santpau.cat (L.A.S.); EFernandezPe@santpau.cat (E.F.P.); dabitarza@gmail.com (D.A.); 5 Cardiovascular Institute, Hospital Clínico San Carlos, IdISSC, 28040 Madrid, Spain; luisnombela@yahoo.com (L.N.-F.); patropjq@gmail.com (P.J.-Q.); albertutor@hotmail.com (J.A.D.A.); ipascualcalleja@yahoo.es (G.T.-C.); 6 Cardiology Department, Reina Sofía University Hospital, University of Córdoba (IMIBIC), 14004 Córdoba, Spain; manuelpanalvarez@gmail.com (M.P.); loladoctora@gmail.com (D.M.); mromero@grupocorpal.com (M.R.); 7 CIBERCV, Cardiology Department, Hospital Clínico Universitario de Valladolid, 47003 Valladolid, Spain; aserradorf@gmail.com (A.S.F.); tania.rdgz.gabella@gmail.com (T.R.-G.); ijamat@gmail.com (I.J.A.-S.); 8 Cardiology Department, Institut Clínic Cardiovascular, Hospital Clinic de Barcelona, 08036 Barcelona, Spain; xavierfreixa@hotmail.com (X.F.); anderregueiro@gmail.com (A.R.); masabate@clinic.cat (M.S.); 9 Cardiology Department, Complejo Hospitalario Universitario de Santiago de Compostela, CIBERCV, 15706 Santiago de Compostela, Spain; ramirotrillo@mac.com (R.T.-N.); hernandezdaniel@uniovi.es (A.M.M.); belcid77@hotmail.com (A.B.C.A.); 10 Cardiology Department, Ramon y Cajal University Hospital, 28034 Madrid, Spain; rhernandez_antolin@hotmail.com (R.A.H.-A.); luisasalido@hotmail.com (L.S.T.); Recalde@secardiologia.es (A.S.-R.); 11 Cardiology Department, Hospital Universitario de Basurto, 48013 Bilbao, Spain; Leire.andrakaikazuriaga@osakidetza.eus (L.A.I.); lararuiz7@hotmail.com (L.R.G.); 12 Cardiology Department, University Hospital of Salamanca, IBSAL, Institute of Biomedical Research of Salamanca, University of Salamanca, CIBERCV, 37007 Salamanca, Spain; cruzgonzalez.ignacio@gmail.com (I.C.-G.); treejooblanca@hotmail.com (B.T.-V.); 13 Cardiology Department, University Hospital of Badajoz, 06080 Badajoz, Spain; lopez-minguez@hotmail.com; 14 Cardiology Department, Hospital Universitario y Politécnico La Fe, 46026 Valencia, Spain; diez_jlu@gva.es; 15 Cardiology Department, Hospital General Universitario de Valencia, 46014 Valencia, Spain; berenguer_alb@gva.es; 16 Cardiology Department, University Clinic Hospital of Valencia, University of Valencia, INCLIVA, CIBERCV, 46010 Valencia, Spain; sanchis_juafor@gva.es; 17 Cardiology Department, Complejo Hospitalario de Navarra, 31008 Pamplona, Spain; valeriano.ruiz.quevedo@navarra.es; 18 Cardiology Department., Complejo Hospital Regional Universitario de Málaga, 29010 Málaga, Spain; cristobalurbano@gmail.com; 19 Cardiology Department, University Hospital Puerta de Hierro/Majadahonda, 28222 Madrid, Spain; jfoteod@hotmail.com; 20 Cardiology Department, University Hospital Miguel Servet, 50009 Zaragoza, Spain; charortasnadal@gmail.com; 21 Cardiology Department, University Hospital Virgen de las Nieves, 18014 Granada, Spain; cardiomol03@gmail.com; 22 Cardiology Department, Hospital Universitari Germans Trias i Pujol, 08916 Barcelona, Spain; xcarrillosuarez@gmial.com; 23 Cardiology Department, Alvaro Cunqueiro University Hospital, 36213 Vigo, Spain; roiestevez@hotmail.com

**Keywords:** Mitraclip, functional mitral regurgitation, transcatheter, left ventricular ejection Fraction

## Abstract

Background: Transcatheter mitral valve repair (TMVR) could improve survival in functional mitral regurgitation (FMR), but it is necessary to consider the influence of left ventricular ejection fraction (LVEF). Therefore, we compare the outcomes after TMVR with Mitraclip^®^ between two groups according to LVEF. Methods: In an observational registry study, we compared the outcomes in patients with FMR who underwent TMVR with and without LVEF <30%. The primary endpoint was the combined one-year all-cause mortality and unplanned hospital readmissions due to HF. The secondary end-points were New York Heart Association (NYHA) functional class and mitral regurgitation (MR) severity. Propensity-score matching was used to create two groups with the same baseline characteristics, except for baseline LVEF. Results: Among 535 FMR eligible patients, 144 patients with LVEF <30% (group 1) and 144 with LVEF >30% (group 2) had similar propensity scores and were included in the analyses. The primary study endpoint was significantlly higher in group 1 (33.3% vs. 9.4%, *p* = 0.002). There was a maintained improvement in secondary endpoints without significant differences among groups. Conclusion: FMR patients with LVEF <30% treated with MitraClip^®^ had higher mortality and readmissions than patients with LVEF ≥30% treated with the same device. However, both groups improved the NYHA functional class and MR severity.

## 1. Introduction

Mitral regurgitation (MR) has become the most common valvular disease. In fact, up to 1 in every 10 individuals aged 75 years or older present moderate or severe MR [1]. The etiology of MR can be either degenerative or functional. Unlike degenerative MR (DMR), in functional mitral regurgitation (FMR) the components of the mitral apparatus are preserved. Thus, FMR is defined as the mitral insufficiency with a lack of leaflet coaption due to annular dilatation or left ventricular (LV) remodeling [2,3].

In contrast to DMR, where mitral valve surgery can improve the prognosis [4,5,6,7], surgical treatment for FMR has not shown to improve functional status or survival [8,9]. Therefore, invasive treatment (either surgical or percutaneus) may be considered in those patients with chronic severe FMR that remain symptomatic despite optimal medical management, when revascularization is not indicated [6,7].

Over the past few years, transcatheter mitral “edge to edge” valve repair (TMVR) using MitraClip^®^ (Abbott Vascular, Menlo Park, CA, USA) system has emerged as a safe and effective treatment option for both high-risk DMR and FMR [10,11,12].

The latest evidence in the treatment of FMR, which classically has had a very poor prognosis and no specific treatment, has placed this entity in the frontline of clinical debates. Whereas the first two randomized control trials for FMR—comparing TMVR plus medical therapy versus medical therapy alone—confirmed a high rate of procedural success, different clinical results in follow-up were found between both studies [13,14]. The patients in both studies have different baseline characteristics. Therefore, finding the key variable that predicts a good result is of the utmost importance [15].

The presence of significant FMR in heart failure patients has been associated with increased morbidity and mortality [16,17,18]. Although correction through percutaneous repair can improve survival significantly, the greatest controversy remains around the time of the intervention. If it is carried out in very advanced stages of the disease, it may not be effective [13,14,19].

Left ventricular ejection fraction (LVEF) is one of the most powerful classic independent predictors of survival in heart failure, as well as one of the variables on which the indication of treatment for both degenerative and functional mitral insufficiency depends on [6,7,20]. Even though FMR has been considered not only a marker but also an independent risk factor for adverse events, it is necessary to take into account the influence of LVEF in this context [18,21].

The cut-off point, LVEF = 30%, is the limit indicated by the guidelines to predict the outcome after surgical repair. In line with the surgical point of view in the guidelines, (LVEF) below 30% could also modify the outcome in TMVR [6,7].

Therefore, our aim was to analyze the differences in one-year all-cause mortality and unplanned hospital readmissions due to heart failure in a cohort of FMR patients treated by TVMR according to their LVEF.

## 2. Methods

We performed our study using a registry-based analysis involving patients with severe FMR patients who underwent TMVR using MitraClip^®^.

Data were obtained from the Spanish MitraClip Registry. This registry is a contemporary prospective clinical-practice registry, and it was endorsed by the Interventional Cardiology Association of the Spanish Society of Cardiology and prospectively included consecutive patients treated with MitraClip from 1 June 2012, to 1 March 2020, from 23 Spanish hospitals. The indication for TMVR was established after multidisciplinary team evaluation (Heart Team) in each center.

A specialized centralized database was designed for the prospective and consecutive inclusion of all of the patients’ demographic, echocardiographic, procedural, and follow-up variables.

All included patients signed a dedicated informed consent form. This study was approved by the local Ethical Committee (reference 2020/026).

### 2.1. Study Population

For the purpose of this study, we included all patients in the registry with severe FMR treated with TMVR using MitraClip^®^ (Abbott; Menlo Park, CA, USA) [3,6,7].

FMR etiology was defined as the one shows structurally normal leaflets and chordae but an imbalance between the closing and tethering forces in the valve, secondary to alterations in left ventricular geometry. Degenerative and Mixed MR etiologies were excluded [3,6,7].

In all participant centers, patients with moderate-severe or severe (3 to 4+) FMR, symptomatic despite guideline-directed optimal medical therapy, were evaluated by a multidisciplinary Heart Team (comprising interventional cardiologists, cardiac surgeons, heart failure specialized cardiologists and cardiac imaging specialists) [6,7]. Informed consent for the procedure was obtained from all patients and TMVR was performed with the MitraClip^®^ edge to edge technique as reported elsewhere [22].

To understand the differences in mortality and unplanned hospital readmissions due to heart failure according to their LVEF, the sample was divided into two groups according to LVEF. Group 1 was composed by patients with severely impaired LVEF (LVEF less to 30%) and group 2 by patients with LVEF ≥30%. Both groups were prospectively followed-up. There were no losses in follow-up.

### 2.2. Variable Definitions

Procedural success was defined as the proper implantation of at least one clip and reduction of the severity of the MR to a grade less than or equal to moderate (2+). The severity of MR, not only for the diagnosis but also for the follow-up, was evaluated by experts cardiac imaging specialists, according to the European Society of Cardiology (ESC) guideline criteria [6,7].

Procedural time was defined as the duration from anesthetic induction to the end of the procedure. Device implantation time was calculated from the insertion of the release system until its removal.

Procedure-related bleeding and its severity were defined according to the criteria of the Bleeding Academic Research Consortium (BARC) [23].

Functional class was defined according to the classification of the New York Heart Association (NYHA).

### 2.3. Study Outcomes

The primary endpoints of the study were (1) the combined 1-year all-cause mortality and unplanned hospital readmissions due to HF, (2) 1-year all-cause mortality and (3) unplanned hospital readmissions due to HF. 

The secondary end-points were (1) functional class after TMVR and (2) MR severity after 1-year follow up.

### 2.4. Statistical Analysis 

Absolute (*n*) and relative (%) frequencies were calculated for qualitative variables. For quantitative variables, Kolmogorov–Smirnov test was used to assess the normality of the variables. Quantitative variables were expressed as mean ± standard deviation if normally distributed and as median (interquartile range) if not. The differences in the qualitative variables were calculated as a percentage difference with the Pearson chi-square test; if 20% or more of cells had expected frequencies <5, likelihood ratio correction was performed.

Propensity score matching was used to create two groups with the same baseline characteristics. We estimated propensity scores and matched for LVEF groups (<30% or ≥30%), using nearest-neighbor matching without replacement. The propensity score is a conditional probability of having a particular exposure given a set of baseline measured covariates. Covariates were chosen based on theorical knowledge: age (stratified by intervals of 10 years: <60; 60–69; 70–79; ≥80), sex, BMI (stratified <25 kg/m^2^, 25–29.99 kg/m^2^, and ≥30 kg/m^2^), hypertension, diabetes mellitus and previous ischemic heart disease.

The matching ratios for the order of formation LVEF groups were 1:1. After the matching, both groups were confirmed to be similar in baseline characteristics using mean standardized differences, which has proved to be the best way, since it does not depend on sample size. Then, outcomes were compared among the groups [24,25].

To evaluate one-year mortality and unplanned readmissions for HF between both groups, Kaplan–Meier survival estimator was used. In conjunction with the stratified log-rank test, the median survival and the survival curves were used to compare the event-free survival rates among the groups. Differences in other quantitative variables were compared with the one-way ANOVA (using post-hoc Bonferroni analysis for multiple comparisons) or the Kruskal–Wallis test according to the distribution of the variable. Differences in other qualitative variables were compared with the Pearson chi-square test.

For data analysis, the SPSS version 23.0 statistical package was used (IBM Corp.; Armonk, New York, NY, USA).

## 3. Results

### Study Population

**Figure 1:**
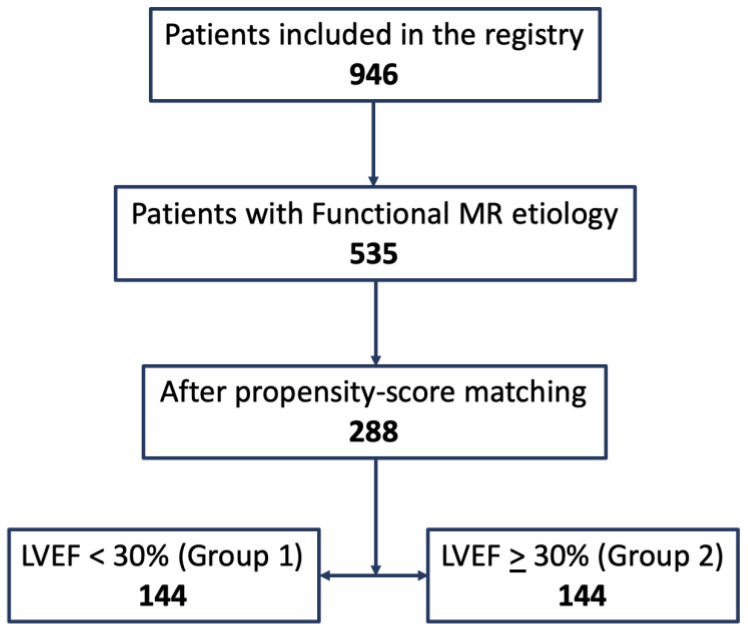
Flow chart of the design of the study.

During the study period, there were 946 patients included in the registry and available for analysis. We identified 535 patients with FMR who met the inclusion criteria (Figure 1), of whom a total of 396 (74%) were men and 139 (26%) were women. The mean age was 71.0 ± 10.8 years old, with an average BMI of 27.2 ± 4.5 kg/m^2^.

In the global series, before propensity score matching, there were significant differences between the two groups in several of the baseline variables (Table 1).

Values represent *n* (%), mean ± standard deviation or median (interquartile range).

With the use of the propensity score, 144 patients who underwent TMVR with a LVEF <30% (group 1) were matched with 144 LVEF ≥30% (group 2) (global matched group 288, Table S1 supplementary material). The flow chart of the design of the study is shown in Figure 1.

After matching, the mean standardized differences were less than 10% for all variables, indicating marginal differences between the two groups (Table 2).

Values represent *n* (%), mean ± standard deviation or median (interquartile range).

There were no significant differences among groups regarding the variables associated with the procedure (Table 3).

## 4. Outcomes

### 4.1. Primary Endpoint

The number of events for the primary study endpoint at 12 months of follow-up in group 1 was 48 (33.3) and 28 (19.4) in group 2, and the difference among both groups was statistically significant (*p* = 0.002). The number of events for the combined endpoint at 12 moths in the global matched group was 76 (26.4%).

The all-cause mortality was distributed according to LVEF as follows: 25 patients (17.4%) in group 1 and 10 patients in group 2 (6.9%). Significant differences were found among both groups for this outcome (*p* = 0.005). The all-cause mortality in the global matched group was 12.2% (35 patients). There were 27 (9.4%) patients who died due to cardiac causes and four (1.4) of them suffered arrhythmic death. There were 8 (2.8%) non-cardiac deaths during the follow up (three Sepsis, three Cancer, one multiorganic failure and one suicide). The causes of death across the groups are detailed in Table 4.

The proportion of unplanned readmissions for HF was 18.4% in the global matched group. The proportion as 20.8% in the in group 1 (30 patients), whereas it was 16.0% (23 patients) in group 2 (*p* = 0.114).

**Figure 2:**
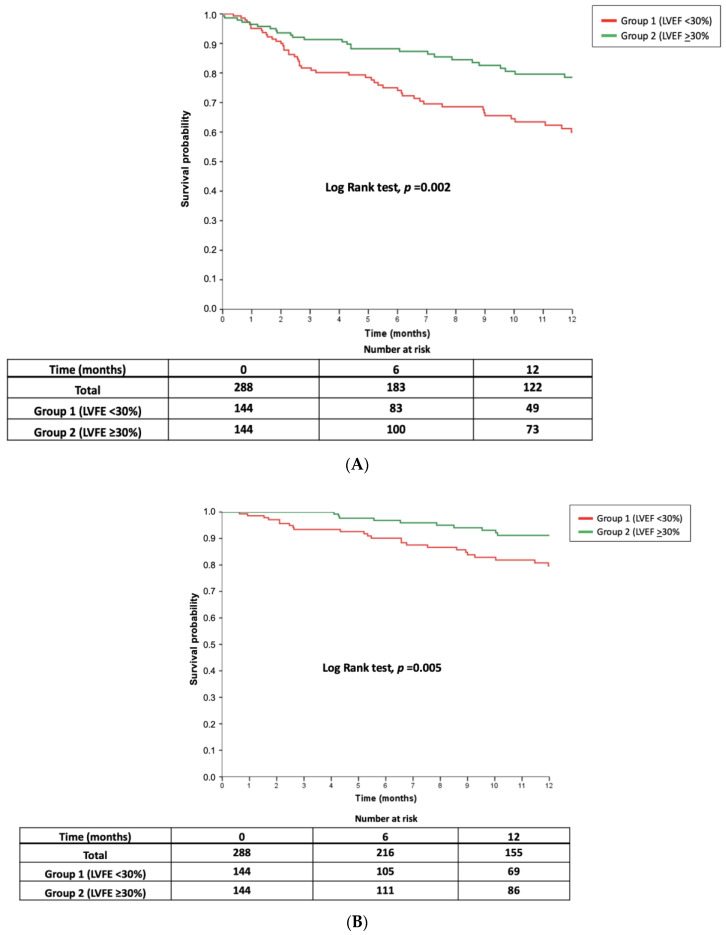

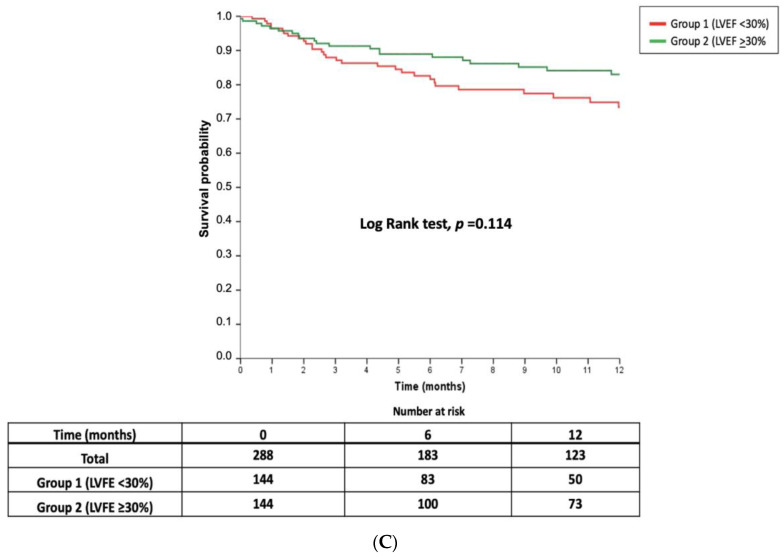
Twelve-month survival curves for the composite event (**A**) Composite event (death/unplanned hospitalization), all-cause mortality. (**B**) All-cause mortality and readmission for heart failure (**C**) Readmission for heart failure according to LVEF groups.

Median time to first readmission was 2.7 (1.5–6.0) in group 1 and 2.8 (1.2–7.3) in group 2 (*p* = 0.921). Figure 2 shows the 12-month survival curves for the composite endpoint (Figure 2A), all-cause mortality (Figure 2B), and readmissions for HF (Figure 2C), according to LVEF.

### 4.2. Secondary Endpoints

**Figure 3:**
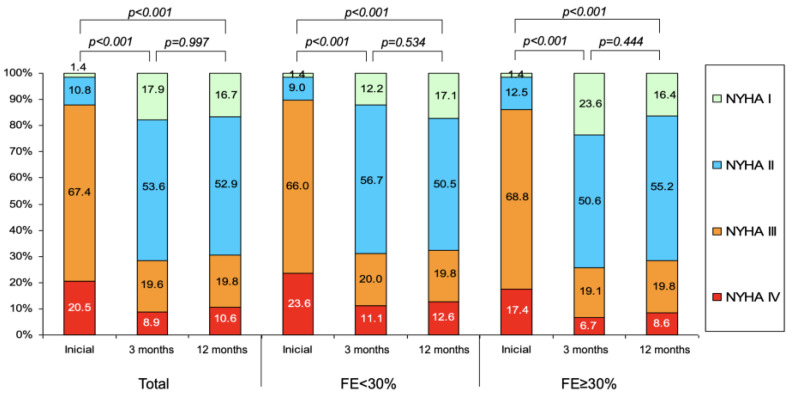
Modifications over time in NYHA functional class during follow-up in the entire series and according to LVEF. NYHA, New York Heart Association.

Changes over time in the NYHA functional class are shown in Figure 3. There was a clear improvement at three months. This was maintained at 12 months of follow-up in both groups. At the end of follow-up, the proportion of patients in class ≤ NYHA II was 67.6% in the group 1 and 71.6% in the group 2, without significant differences among them (*p* = 0.774).

**Figure 4:**
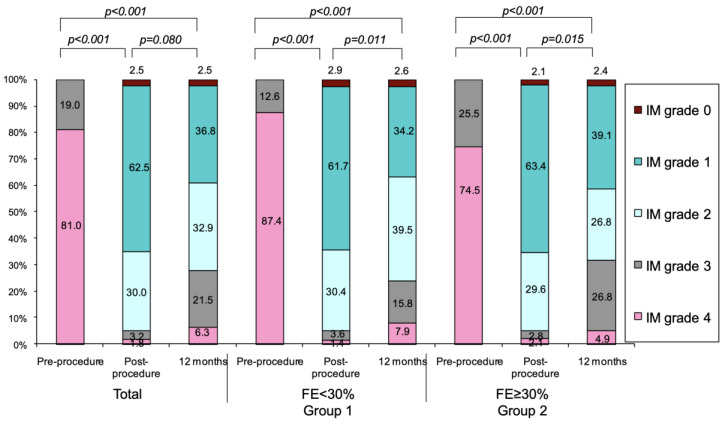
Changes over time in mitral regurgitation (MR) grade during follow-up in the entire series and according to LVEF groups. MR, mitral regurgitation.

Regarding MR reduction (Figure 4), there was a clear improvement after the procedure, and it was maintained at one year. At the end of follow up, 76.3% of the patients in group 1 and 68.2% in group 2 had less or equal grade 2+ MR (*p* = 0.643). During the follow up, four patients (one patient (0.7%) in group 1 and three patients (2.1%) in group 2, *p* = 0.622) underwent conventional mitral valve surgery.

## 5. Discussion

Our study highlights the relevance of severe LV dysfunction as a variable associated with death and rehospitalizations in a cohort of patients with FMR treated by percutaneous repair. LVEF should be a key element to analyze when selecting patients for this strategy. However, in spite of severe LV dysfunction, our paper shows that patients can still improve their functional clinical status. This is relevant also when dealing with this complex population, who are often short of therapeutic alternatives.

In the current study, we show that patients with FMR and severe LV dysfunction treated with MitraClip^®^ have higher combined mortality and readmissions than patients without severe LV dysfunction treated with the same device. However, these patients showed no significant differences in the unplanned HF rehospitalization. Both groups showed significant improvement in the degree of MR and in the functional NYHA class. Our findings are in the line with previous registries regarding the prognosis implication of LVEF in patients treated with TMVR [10,11].

The association between the severity of left ventricular dysfunction and clinical outcomes following intervention with MitraClip^®^ was established using a meta-regression in a recent metanalysis, partially explaining the contradicting results observed in the COAPT and the MITRA-FR trial [26].

Results from other interesting metanalyses highlighted the possible association between LV impairment and relative risk of all-cause and cardiovascular mortality, suggesting that patients with poor LVEF probably benefit less from TVMR [27].

The global clinical improvement after Mitraclip^®^ in FMR remains under permanent study. While two recent observational studies showed worse outcomes in patients with lower LVEF [28,29], other reports have recently showed that the treatment with MitraClip^®^ for FMR in patients with different degrees of LV dysfunction is associated with a considerable reduction of death and HF hospitalization at mid-term follow-up [30].

Both groups of our study had significant improvements after TVMR with Mitraclip^®^ in the NYHA functional class and reduction in the grade of MR at the end of the follow-up. These results agree with previous reports of multinational real-life registries, where high rates of sustained MR reduction and clinical benefit were found also in patients with impaired LVEF [10,11,12,31]. Moreover, this could be a relevant point to take into account in order to consider the value of TMVR, not only for the improvement of prognosis in not-advanced stages of the disease, but also for the improvement in the quality of life of these patients in the follow up [32,33].

According to the results of our study, the prognostic implication of severe LV dysfunction in FMR patients should be considered in the selection of candidates for TMVR. In order to improve the prognosis of our patients, we should consider anticipating the treatment before severe deterioration in LVEF, as it was shown in the clinical trials [13,14]. However, we have to take into account that the severe impairment of LVEF should not contraindicate TMVR with Mitraclip^®^, because of the improvement in functional class and quality of life obtained in these symptomatic patients.

## 6. Limitations

Some limitations of our analysis should be considered. This was a non-randomized, observational study, and hence, it suffers from potential selection and ascertainment bias, despite robust propensity score matching. It is possible that some patients were lost on follow-up; however, this is an inherent limitation of all observational studies and we tried compensate this fact with a thorough clinical and echographic follow-up of all patients.

## 7. Conclusions

The careful selection of patients with FMR may be the most critical factor to predict favorable outcomes with the MitraClip^®^ device. Therefore, it is very important to identify patients who could really benefit from TVMR in terms of prognosis. LVEF could be one of the most important variables. Patients with severe LV dysfunction treated with MitraClip^®^ have higher mortality and readmissions than patients without severe dysfunction when treated with the same device. However, both groups obtain functional-class clinical benefit.

## Figures and Tables

**Table 1:** Baseline characteristics in the global series and among both groups according to the left ventricular ejection fraction (LVEF).

Variable	Total FMR Group *n* = 535	FE < 30% *n* = 229 (42.8%)	FE ≥ 30% *n* = 306 (57.2%)	*p*
Age (years)	71.0 ± 10.8	68.1 ± 11.7	73.2 ± 9.7	<0.001
Sex (*n* (%)) Men Women	396 (74.0) 139 (26.0)	174 (76.30) 55 (24.0)	222 (72.5) 84 (27.5)	0.370
BSA (m^2^)	1.82 ± 0.20	1.83 ± 0.23	1.76 ± 0.38	0.012
BMI (kg/m^2^)	27.2 ± 4.5	26.6 ± 4.4	27.6 ± 4.5	0.050
BMI ≥ 30 kg/m^2^	124 (23.2)	44 (19.2)	80 (26.1)	0.060
LVEF, %	34.3 ± 12.5	23.5 ± 6.4	42.1 ± 9.9	-
Type 2 Diabetes Mellitus	189 (35.3)	79 (34.5)	110 (35.9)	0.728
Ischemic Heart Disease	302 (56.4)	115 (50.2)	187 (61.1)	0.012
Hypertension	372 (69.5)	140 (61.1)	232 (75.8)	<0.001
Previous Cardiac Surgery	89 (16.6)	30 (13.1)	59 (19.3)	0.058
Hemodyalisis	9 (1.7)	3 (1.3)	6 (2.0)	0.739
Creatinine (mg/dL)	1.37 ± 0.57	1.34 ± 0.51	1.40 ± 0.61	0.268
eGFR (mL/min)	62.2 ± 25.8	62.8 ± 23.2	61.7 ± 27.6	0.763
NYHA Class I II III IV	12(2.2) 59(11.0) 355 (66.4) 109 (20.4)	4(1.7) 22(9.6) 149 (65.1) 54 (23.6)	8(2.6) 37(12.1) 206 (67.3) 55 (18.0)	0.348
STS Score	3.7 (1.8–6.7)	3.2 (1.6–6.4)	3.6 (2.1–6.8)	0.135
Active Endocarditis	4 (0.7)	1 (0.4)	3 (1.0)	0.639
TAPSE	14.9 ± 6.7	15.6 ± 5.5	14.4 ± 7.3	0.109
Dyslipidemia	305 (57.0)	131 (57.2)	174 (56.9)	0.937
Critical Preoperative Status	28 (5.2)	16 (7.0)	12 (3.9)	0.115
Extracardiac Arteriopathy	73 (13.6)	32 (14.0)	41 (13.4)	0.848
Unstable Angina	16 (3.0)	7 (3.1)	9 (2.9)	0.938
Permanent Atrial Fibrilation	313 (58.5)	123 (53.7)	190 (62.1)	0.052
Urgent Cardiac Surgery	49 (9.2)	19 (8.3)	30 (9.8)	0.550
Smoker	154 (28.8)	59 (25.8)	95 (31.0)	0.182
COPD	111 (20.7)	45 (19.7)	66 (21.6)	0.588
Recent Myocardial Infarction	41 (7.7)	17 (7.4)	24 (7.8)	0.857
Permanent Pacemaker	74 (13.8)	32 (14.0)	42 (13.7)	0.934
Stroke	58 (10.8)	26 (11.4)	32 (10.5)	0.741
Percutaneous revacularization	210 (39.3)	85 (37.1)	125 (40.8)	0.382
CABG	88 (16.4)	23 (10.0)	65 (21.2)	0.001
Cardiac Resyncronization	83 (15.5)	51 (22.3)	32 (10.5)	<0.001
Autoimplantable cardiodefibrilator	178 (33.3)	109 (47.6)	69 (22.5)	<0.001
Prior TAVI	13 (2.4)	7 (3.1)	6 (2.0)	0.415
Poor Morbility	52 (9.7)	23 (10.0)	29 (9.5)	0.827
Previous Heart Transplantation	29 (5.4)	14 (6.1)	15 (4.9)	0.540
Prior Mitral Annuloplasty	10 (1.9)	3 (1.3)	7 (2.3)	0.528
Aortic Surgery	15 (2.8)	3 (1.2)	12 (3.9)	0.070
Technical Procedural Success	502 (93.8)	214 (93.4)	288 (94.1)	0.751

BMI, body mass index; BSA, Body surface area; CABG, coronary artery bypass graft; COPD, chronic obstructive pulmonary disease; eGFR, estimated glomerular filtration rate; LVEF, left ventricular ejection fraction; NYHA, New York Heart Association; TAVI, transcatheter aortic valve implantation. Recent MI is defined as happening between 7 and 30 days ago. Poor morbidity was an indirect measure of frailty based on the medical history (slowness, weakness, exhaustion, wasting and malnutrition, poor endurance and inactivity or loss of independence).

**Table 2:** Baseline characteristics of propensity score matched group and among both groups according to the LVEF.

	Matched Group *n* = 288	FE < 30% (Group 1) *n* = 144	FE ≥ 30% (Group 2) *n* = 144	*p*
Age (years)	71.5 ± 9.8	71.3 ± 10.5	71.8 ± 9.1	0.676
Sex (*n* (%)) Men Women	228 (79.2) 60 (20.8)	114 (79.2) 30 (20.8)	114 (79.2) 30 (20.8)	-
BSA (m^2^)	1.75 ± 0.36	1.79 ± 0.19	1.71 ± 0.48	0.140
BMI (kg/m^2^)	26.5 ± 4.2	26.4 ± 4.0	26.6 ± 4.3	0.736
BMI ≥ 30 kg/m^2^	54 (18.8)	27 (18.8)	27 (18.8)	-
LVEF, %	32.9 ± 11.8	24.2 ± 5.9	41.6 ± 9.6	-
Type 2 Diabetes Mellitus	96 (33.3)	48 (33.3)	48 (33.3)	-
Ischemic Heart Disease	186 (64.6)	93 (64.6)	93 (64.6)	-
Hypertension	204 (70.8)	102 (70.8)	102 (70.8)	-
Previous Cardiac Surgery	53 (18.4)	25 (17.4)	28 (19.4)	0.648
Hemodyalisis	7 (2.4)	2 (1.4)	5 (3.5)	0.447
Creatinine (mg/dL)	1.39 ± 0.61	1.38 ± 0.49	1.41 ± 0.71	0.643
eGFR (mL/min)	65.1 ± 29.0	63.0 ± 24.8	67.1 ± 32.6	0.433
NYHA Class I II III IV	5 (1.7) 28 (9.7) 190 (66.0) 65 (22.6)	1 (0.7) 11 (7.6) 93 (64.6) 39 (27.1)	4 (2.8) 17 (11.8) 97 (67.4) 26 (18.1)	0.115
STS Score	3.9 (1.3–6.8)	3.7 (1.9–7.2)	3.5 (1.5–6.5)	0.511
Active Endocarditis	1 (0.3)	0	1 (0.7)	1
TAPSE	14.7 ± 6.8	15.1 ± 6.3	14.8 ± 7.7	0.274
Dyslipidemia	169 (58.7)	88 (61.1)	81 (56.3)	0.402
Critical Preoperative Status	13 (4.5)	8 (5.6)	5 (3.5)	0.394
Extracardiac Arteriopathy	46 (16.0)	21 (14.6)	25 (17.4)	0.520
Unstable Angina	10 (3.5)	5 (3.5)	5 (3.5)	1
Permanent Atrial Fibrilation	163 (56.6)	80 (55.6)	83 (57.6)	0.721
Urgent Cardiac Surgery	22 (7.6)	9 (6.3)	13 (9.0)	0.375
Smoker	83 (28.8)	43 (29.9)	40 (27.8)	0.696
Chronic obstructive pulmonary disease	56 (19.4)	29 (20.1)	27 (18.8)	0.766
Recent Myocardial Infarction	22 (7.6)	10 (6.9)	12 (8.3)	0.825
Permanent Pacemaker	44 (15.3)	22 (15.3)	22 (15.3)	1
Stroke	34 (11.8)	18 (12.5)	16 (11.1)	0.715
Percutaneous revacularization	129 (44.8)	67 (46.5)	62 (43.1)	0.554
CABG	54 (18.8)	20 (13.9)	34 (23.6)	0.035
Cardiac Resyncronization	42 (14.6)	26 (18.1)	16 (11.1)	0.095
Autoimplantable Cardiodefibrilator	91 (31.6)	61 (42.4)	30 (20.8)	<0.001
Prior TAVI	8 (2.8)	6 (4.2)	2 (1.4)	0.282
Poor Morbility	26 (9.0)	13 (9.0)	13 (9.0)	1
Previous Heart Transplantation	14 (4.9)	7 (4.9)	7 (4.9)	1
Prior Mitral annuloplasty	4 (1.4)	2 (1.4)	2 (1.4)	1
Surgery Aorta	7 (2.4)	2 (1.4)	5 (3.5)	0.447
Technical Procedural Success	271 (94.1)	135 (93.8)	136 (94.4)	0.803

BMI, body mass index; BSA, Body surface area; CABG, coronary artery bypass graft; COPD, chronic obstructive pulmonary disease; eGFR, estimated glomerular filtration rate; LVEF, left ventricular ejection fraction; NYHA, New York Heart Association; TAVI, transcatheter aortic valve implantation. Recent MI is defined as happening between 7 and 30 days ago. Poor morbidity was an indirect measure of frailty based on the medical history (slowness, weakness, exhaustion, wasting and malnutrition, poor endurance and inactivity or loss of independence).

**Table 3:** Procedure-related variables.

	Matched Group *n* = 288	FE < 30% (Group 1) *n* = 144	FE ≥ 30% (Group 2) *n* = 144	*p*
Procedural success, *n* (%)	271 (94.1)	135(93.8)	136 (94.4)	0.803
Number of clips implanted, *n*	1.49 ± 0.64	1.48 ± 0.64	1.50 ± 0.64	0.744
Procedural duration, min	140 (115–180)	141 (118–192)	136 (105–179)	0.413
Implantation time, min	80 (60–107)	82 (59–108)	70 (60–108)	0.677
Degree of mitral regurgitation post clip, *n* (%) 0 I II III IV	17 (5.9) 176 (61.1) 81 (28.1) 8 (2.8) 6 (2,1)	9 (6.3) 86 (59.7) 41 (28.5) 5 (3.5) 3 (2.1)	8 (5.6) 90 (62.5) 40 (27.8) 3 (2.1) 3 (2.1)	0.955
Transmitral gradient after the clip, mmHg	3.05 ± 1.68	3.06 ± 1.49	3.04 ± 1.84	0.923
Clip detachment (partial or complete),	8 (2.8)	5 (3.5)	3 (2.1)	0.723
Catheter thrombosis, *n* (%)	2 (0.7)	1 (0.7)	1 (0.7)	1
Subvalvular chordal rupture *n* (%)	3 (1.0)	1 (0.7)	2 (1.4)	0.999
Clip entanglement in subvalvular apparatus, *n* (%)	0	0	0	-
Puncture site hematoma, *n* (%)	10 (3.5)	5 (3.5)	5 (3.5)	1
Arteriovenous fistula, *n* (%)	3 (1.0)	2 (1.4)	1 (0.7)	0.999
Valvular surgery, *n* (%)	1 (0.3)	0	1 (0.7)	0.999
Hemorrhage (BARC criteria) 0 1 2 3a 3b	250 (86.8) 32 (11.1) 2 (0.7) 1 (0.3) 3 (1.0)	133 (92.4) 9 (6.3) 1 (0.7) 0 1 (0.7)	117 (81.3) 23 (16.0) 1 (0.7) 1 (0.7) 2 (1.4)	0.590
Pericardial leak	5 (1.7)	3 (2.1)	2 (1.4)	0.685
Urgent indication, *n* (%)	19 (6.6)	10 (6.9)	9 (6.3)	0.812

Values represent *n* (%), mean ± standard deviation or median (interquartile range).

**Table 4:** Causes of death.

	Matched Group *n* = 288	FE < 30% (Group 1) *n* = 144	FE ≥ 30% (Group 2) *n* = 144	*p*
Deaths	35 (12.2)	25 (17.4)	10 (6.9)	0.007
Cardiac death	27 (9.4)	20 (13.9)	7 (4.9)	0.009
Arrhythmic death	4 (1.4)	3 (2.1)	1 (0.7)	0.622
Non cardiac death	8 (2.8)	5 (3.5)	3 (2.1)	0.723

Median time to death was 5.5 (2.3–9.0) months in group 1 and 7.2 (4.3–9.7) months in group 2 (*p* = 0.333).

## Supplementary Materials

The following are available online at https://www.mdpi.com/2077-0383/9/6/1792/s1, Table S1. Baseline characteristics of global FMR group and propensity score matched group.
