# Supplementary material for: Transcatheter Mitral Repair for Functional Mitral Regurgitation According to Left Ventricular Function: A Real-Life Propensity-Score Matched Study
Source: J Clin Med. 2020 Jun 9;9(6):1792. doi: 10.3390/jcm9061792 (PMC7356666; doi:10.3390/jcm9061792)
Supplement: Supplementary file 1 [file jcm-09-01792-s001.pdf]

Table S1. Baseline characteristics of global FMR group and propensity score matched group.

Variable	Total FMR sample $n = 535$	Matched group $n = 288$	p
Age (years)	71.0 ± 10.8	71.5 ± 9.8	0.513
Sex (n(%)			
Men	396(74.0)	228(79.2)	0.100
Women	139(26.0)	60(20.8)	
BSA (m^2)	1.82 ± 0.20	1.75 ± 0.36	<0.001
IMC (Kg/m^2)	27.2 ± 4.5	26.5 ± 4.2	0.030
IMC $\geq$30 Kg/m^2	124(23.2)	54(18.8)	0.141
LVEF, %	34.3 ± 12.5	32.9 ± 11.8	0.119
Type 2 Diabetes Mellitus	189(35.3)	96(33.3)	0.566
Ischemic Heart Disease	302(56.4)	186(64.6)	0.024
Hypertension	372(69.5)	204(70.8)	0.698
Previous Cardiac Surgery	89(16.6)	53(18.4)	0.522
Hemodyalisis	9(1.7)	7(2.4)	0.458
NYHA Class			
I	12(2.2)	5(1.7)	
II	59(11.0)	28(9.7)	0.810
III	355(66.4)	190(66.0)	
IV	109(20.4)	65(22.6)	
STS Score	3.7(1.8–6.7)	3.9(1.3–6.8)	0.846
Active Endocarditis	4(0.7)	1(0.3)	0.663
Dyslipidemia	305(57.0)	169(58.7)	0.644
Critical Preoperative	28(5.2)	13(4.5)	0.651
Extracardiac Arteriopathy	73(13.6)	46(16.0)	0.365
Unstable Angina	16(3.0)	10(3.5)	0.706
Atrial Fibrilation	313(58.5)	163(56.6)	0.597
Urgent Cardiac Surgery	49(9.2)	22(7.6)	0.459
Smoker	154(28.8)	83(28.8)	0.992
Chronic Obstructive pulmonary disease	111(20.7)	56(19.4)	0.658
Recent Myocardial Infarction	41(7.7)	22(7.6)	0.990
Permanent Pacemaker	74(13.8)	44(15.3)	0.572
Stroke	58(10.8)	34(11.8)	0.675
Percutaneous revacularization	210(39.3)	129(44.8)	0.124
CABG	88(16.4)	54(18.8)	0.405
Cardiac Resyncronization	83(15.5)	42(14.6)	0.723
Prior TAVI	13(2.4)	8(2.8)	0.763
Poor Morbility	52(9.7)	26(9.0)	0.747
Previous Heart Transplantation	29(5.4)	14(4.9)	0.731
Prior Mitral annuloplasty	10(1.9)	4(1.4)	0.780
Aortic Surgery	15(2.8)	7(2.4)	0.752
Technical Procedural Success	502(93.8)	271(94.1)	0.879

BMI, body mass index; BSA, Body surface area; CABG, coronary artery bypass graft; COPD, chronic obstructive pulmonary disease; LVEF, left ventricular ejection fraction; NYHA, New York Heart Association; TAVI, transcatheter aortic valve implantation. Values represent *n* (%), mean ± standard deviation or median [interquartile range].
